# Characterization of Phenotypic and Genotypic Diversity of *Stenotrophomonas maltophilia* Strains Isolated From Selected Hospitals in Iran

**DOI:** 10.3389/fmicb.2019.01191

**Published:** 2019-05-29

**Authors:** Narjess Bostanghadiri, Zohreh Ghalavand, Fatemeh Fallah, Abbas Yadegar, Abdollah Ardebili, Samira Tarashi, Abazar Pournajaf, Jalal Mardaneh, Saeed Shams, Ali Hashemi

**Affiliations:** ^1^ Infectious Diseases and Tropical Medicine Research Center, Shahid Beheshti University of Medical Sciences, Tehran, Iran; ^2^ Department of Microbiology, School of Medicine, Shahid Beheshti University of Medical Sciences, Tehran, Iran; ^3^ Foodborne and Waterborne Diseases Research Center, Research Institute for Gastroenterology and Liver Diseases, Shahid Beheshti University of Medical Sciences, Tehran, Iran; ^4^ Infectious Diseases Research Center, Golestan University of Medical Sciences, Gorgan, Iran; ^5^ Department of Microbiology, Faculty of Medicine, Golestan University of Medical Sciences, Gorgan, Iran; ^6^ Department of Mycobacteriology and Pulmonary Research, Pasteur Institute of Iran, Tehran, Iran; ^7^ Department of Microbiology, School of Medicine, Babol University of Medical Sciences, Babol, Iran; ^8^ Microbiology Department, School of Medicine, Gonabad University of Medical Sciences, Gonabad, Iran; ^9^ Cellular and Molecular Research Center, Qom University of Medical Sciences, Qom, Iran

**Keywords:** antibiotic resistance genes, biofilm, efflux pump, sequence type, *Stenotrophomonas maltophilia*, trimethoprim-sulfamethoxazole

## Abstract

*Stenotrophomonas maltophilia* is an environmental Gram-negative bacterium that has rapidly emerged as an important nosocomial pathogen in hospitalized patients. Treatment of *S. maltophilia* infections is difficult due to increasing resistance to multiple antibacterial agents. The purpose of this study was to determine the phenotypic and genotypic characterization of *S. maltophilia* isolates recovered from patients referred to several hospitals. A total of 164 clinical isolates of *S. maltophilia* were collected from hospitals in various regions in Iran between 2016 and 2017. Antibiotic susceptibility testing was performed by disc diffusion method and E-test assay according to the Clinical and Laboratory Standards Institute (CLSI) guideline. The ability of biofilm formation was assessed with crystal violet staining and then, biofilm-associated genes were investigated by PCR-sequencing method. The presence of *L1* (a metallo-β-lactamase), *L2* (a clavulanic acid-sensitive cephalosporinase), *sul1* and *sul2* (resistance to Trimethoprim/Sulfamethoxazole), Sm*qnr* (intrinsic resistance to quinolones), and *dfrA* genes (dihydrofolate reductase enzyme that contributes to trimethoprim resistance) was also examined by PCR-sequencing. Relative gene expression of *smeDEF* efflux pump was assessed by real-time PCR. Genotyping was performed using the multi-locus sequencing typing (MLST) and repetitive extragenic palindromic-PCR (Rep-PCR). Isolates were resistant to imipenem (100%), meropenem (96%), doripenem (96%), and ceftazidime (36.58%). Notably, 5 (3.04%) isolates showed resistant to trimethoprim-sulfamethoxazole (TMP-SMX), an alarming trend of decreased susceptibility to TMP-SMX in Iran. Minocycline and levofloxacin exhibited the highest susceptibility of 91.46 and 99.39%, respectively. Using the crystal violet staining, 157 (95.73%) isolates had biofilm phenotype: 49 (29.87%), 63 (38.41%), and 45 (27.43%) isolates were categorized as strong-, moderate- and weak-biofilm producer while 7 isolates (4.26%) were identified a non-biofilm producer. Biofilm genes had an overall prevalence of 145 (88.41%), 137 (83.53%), and 164 (100%) of *rmlA*, *rpfF*, and *spgM*, respectively. *L1*, *L2*, *Smqnr*, *sul1*, and *sul2* resistance genes were detected in 145 (88.41%), 156 (96.12%), 103 (62.80%), 89 (54.26%), and 92 (56.09%) isolates, respectively. None of the *S. maltophilia* isolates were positive for *dfrA12*, *dfrA17*, and *dfrA27* genes. Gene expression analysis showed that *smeD* efflux system was overexpressed in two out of the five clinical isolates (40%) that showed resistance to TMP-SMX. Most of the isolates were genetically unrelated. Two new sequence types (ST139 and ST259) were determined. Our results showed that TMP-SMX was still an effective antibiotic against *S. maltophilia*. The findings of the current study revealed an increasing prevalence of antibiotic resistance and biofilm genes in clinical *S. maltophilia* isolates in Iran.

## Introduction

The genus *Stenotrophomonas*, together with *Xanthomonas*, belongs to the γ-β subclass of proteobacteria (Anzai et al., 2000). *S. maltophilia* isolated in 1943 from pleural effusion of patients was first named as *Bacterium bookeri*. Later, it was reclassified as a member of the genera *Pseudomonas* and *Xanthomonas* in 1961 and 1983, respectively, until it was classified as a new genus, *Stenotrophomonas*, in 1993 (Al-Anazi and Al-Jasser, 2014).

*S. maltophilia* is a Gram-negative, non-fermentative, aerobic, motile bacillus that is abundant in the ubiquitous environment with a broad geographical distribution. This organism has emerged as an important opportunistic pathogen in humans worldwide. Although it is considered to have limited pathogenicity (Di Bonaventura et al., 2010), *S. maltophilia* causes various types of hospital- and community-acquired *infections,* especially in debilitated or immunocompromised patients, with the mortality rate of 37.5% (Falagas et al., 2009). The bacterium has been increasingly recognized as responsible for a number of clinical syndromes, such as pneumonia, sepsis, bacteremia, endocarditis, septic arthritis, meningitis, endophthalmitis, and urinary infections (Looney et al., 2009; Sumida et al., 2015; Hu et al., 2016).

During the last decade, *S. maltophilia* has been considered as one of the leading multi-drug resistant (MDR) organisms in hospital settings due to exhibiting high levels of intrinsic and acquired resistance to a broad array of antibacterial agents, including fluoroquinolones, aminoglycosides, and the most common of β-lactam antibiotics (Brooke, 2014). Different types of antimicrobial resistance mechanisms, such as expression of antibiotic hydrolyzing or modifying enzymes, membrane permeability alteration (Hu et al., 2008), and multi-drug efflux systems (Huang et al., 2014) have been identified in *S. maltophilia*.

This bacterium produces two chromosomal-mediated inducible β-lactamases, known as *L1* and *L2*. The *L1* belongs to molecular class B Zn^2+^-dependent metallo-β-lactamase (MBL), is resistant to clavulanic acid and hydrolyses carbapenems, cephalosporins, and penicillins (Brooke, 2012; Chang et al., 2015). *The L2 serine-β*-*lactamase*, an *Ambler class* A enzyme, is an inducible cephalosporinase that hydrolyses cephalosporins, penicillins, and aztreonam (Flores-Trevino et al., 2014; Mojica et al., 2016). Two mechanisms are associated with resistance to quinolones among *S. maltophilia* strains, including *smeDEF*, *smeIJK*, *smeABC*, and *smeVWX* efflux pumps and a novel chromosomal quinolone resistance gene, *Smqnr*, encoding the pentapeptide repeat protein that protects both topoisomerase IV and gyrase from the quinolones (Sanchez et al., 2009; Chang et al., 2015; Kanamori et al., 2015).

Trimethoprim-sulfamethoxazole (TMP-SMX) is recommended as the *first choice* for *S*. *maltophilia* infections (Abbott et al., 2011; Chong et al., 2017). However, the increasing reports of resistance to TMP-SMX are a matter of concern and have complicated the treatment strategies (Brooke, 2014; Hu et al., 2016; Madi et al., 2016). Resistance to this antibiotic has been recognized due to the presence of *sul1* and *sul2* genes that are found in class 1 integrons and *insertion sequence common region* (ISCR) elements, respectively. *dfrA* gene cassettes are observed in class 1 integrons and encode for the dihydrofolate reductase enzyme, and TolCsm, smeDEF, smeYZ efflux pumps (Hu et al., 2011, 2016; Huang et al., 2013; Lin et al., 2015; Sánchez and Martínez, 2015).

Biofilms are multicellular communities usually held together by extracellular matrix molecules. These extracellular polysaccharides (EPS) produced by the bacteria usually function as highly organized multicellular communities of microorganisms (Bjarnsholt et al., 2009; Irie et al., 2017), appear to be preferred survival strategy of microbes, and confer tolerance to high doses of antimicrobial agents than non-biofilm forming bacteria (Bjarnsholt et al., 2009). In addition, they are increasingly recognized as a contributing factor in the pathogenesis of disease in respiratory diseases often caused by chronic bacterial infections. *S. maltophilia* strains are well*-*known biofilm-producing organisms with ability to adhere to biotic and abiotic surfaces (Pompilio et al., 2008). Few genes associated with biofilm formation in *S. maltophilia* have been experimentally studied (Liu et al., 2017). More recently, the correlation between mutations in *rpfF* and *rmlA* genes, encoding enoyl-CoA hydratase and glucose-1-phosphate thymidyltransferase, respectively, and the less extensive biofilm formation have been reported (Huang et al., 2006; Fouhy et al., 2007). In addition, the *spgM* gene, responsible for the production of phosphoglucomutase (PGM) and phosphomannomutase, could be involved in biofilm-forming ability (McKay et al., 2003; Zhuo et al., 2014).

High genetic diversity was identified among *S. maltophilia* strains through the use of a variety of molecular biology techniques. Several genotypic profile methods have been used to compare and link clinical isolates to environmental sources, including whole genome sequencing analyses, amplified fragment length polymorphism (AFLP) fingerprinting, PCR*-*restriction fragment length polymorphism (PCR-*RFLP*), analysis of the *gyrase B* gene, PCR-based fingerprinting methods, such as BOX and repetitive extragenic palindromic (rep)-PCR, enterobacterial repetitive intergenic consensus (ERIC)-PCR, pulsed-field gel electrophoresis (PFGE) analysis of *XbaI* genomic digests, and multi-locus sequence typing (MLST) (Gherardi et al., 2015). Rep-PCR technique is based on the fact that microbial genomes contain a variety of repetitive sequences. Although their function has mostly not been elucidated so far, most rep-PCR-based DNA fingerprinting studies have used short polytrinucleotides, such as (GTG)_5_ 35–40 bp repetitive sequences, and 154 bp BOX element as priming sites for PCR, resulting in amplification of DNA sequences between the repetitive parts (Ishii and Sadowsky, 2009). MLST technique was developed for tracking the source of infections and the distribution of pathogens isolated from hospitalized patients, providing reliable epidemiological data. In addition, because of its accessible related international databases, the results from different laboratories by MLST can be compared (Cho et al., 2012).

The main purpose of this study was to evaluate the antimicrobial resistance patterns and different resistance mechanisms of the clinical *S. maltophilia* isolated from different regions of Iran. In addition, the ability of biofilm production as well as clonal and genetic diversity of isolates were examined.

## Materials and Methods

### Ethics Statement

This study was approved by the Ethics Committee of Shahid Beheshti University of Medical Sciences “IR.SBMU. MSP.REC.1397.579.” In order to maintain patients confidentiality participants were anonymous and no personal information was collected or included in the study.

### Bacterial Isolation and Species Identification

*S. maltophilia* isolates were collected from different hospitalized patients in selected hospitals in Iran over a 12-months period from May 2016 to May 2017. Laboratory identification of isolates was carried out using the standard biochemical methods, such as oxidase and catalase tests, and reactions in media, including deoxyribonuclease test agar (Merck Cat. No.1.10449.0500), triple sugar iron agar (Merck Cat. No 1.03915.0500), and SIM (Merck Cat. No1.05470.0500). Consequently, isolates were confirmed as *S. maltophilia* by using the 16S rRNA sequencing with specific primers (Table 1; Kettleson et al., 2013). All isolates were stored in LB with 20% glycerol at −70°C. *Escherichia coli* ATCC 35218, *Pseudomonas aeruginosa* ATCC 27853, *E. coli* ATCC 25922, and *S. maltophilia* ATCC 13637 were used as the quality control strains.

**Table 1:** Oligonucleotide primers used in this study.

Primers	Sequences(5′_3′)	Target	References
*16srRNA-F* *16srRNA-R*	AGTTCGCATCGTTTAGGG ACGGCAGCACAGAAGAGC	16 s RNA	(Di Bonaventura et al., 2010)
*L1-F* *L1-R*	AGCCGTTAAAATTAAGCCC CTTGATTGAAGGGTTGGGCG	L1	(Flores-Trevino et al., 2014)
*L2-F* *L2-R*	CGACAATGCCGCAGCTAACC CAGAAGCAATTAATAACGCCC	L2	(Flores-Trevino et al., 2014)
*Smqnr-F* *Smqnr-R*	ACACAGAACGGCTGGACTGC TTCAACGACGTGGAGCTGT	Smqnr	(Kanamori et al., 2015)
*sul1-F* *sul1-R*	ATGGTGACGGTGTTCGGCATTCTGA CTAGGCATGATCTAACCCTCGGTC	sul1	(Hu et al., 2008)
*sul2-F* *sul2-R*	GAAGCGCAGCCGCAATTCAT CCTGTTTCGTCCGACACAGA	sul2	(Hu et al., 2008)
*spgM-F* *spgM-R*	ATACCGGGGTGCGTTGAC CATCTGCATGTGGATCTCGT	spgM	(Madi et al., 2016)
*rpfF-F* *rpfF-R*	CACGACAGTACAGGGGACC GGCAGGAATGCGTTGG	rpfF	(Madi et al., 2016)
*rmlA-F* *rmlA-R*	CGGAAAAGCAGAACATCG GCAACTTGGTTTCAATCACTT	rmlA	(Madi et al., 2016)
*dfrA12-F* *dfrA12-R*	TTAGCCGTTTCGACGCGCAT ATGAACTCGGAATCAGTACGC	dfrA12	(Hu et al., 2008)
*dfrA17-F* *dfrA17-R*	GTTAGCCTTTTTTCCAAATCTGGTATG TTGAAAATATTATTGATTTCTGCAGTG	dfrA17	(Hu et al., 2008)
*DfrA27-F* *DfrA27-R*	AAGAGTCTGATCGCCCATGCCG TAAAGCAATAACTTACAATC	dfrA27	(Hu et al., 2008)
*SmeD-F* *SmeD-R*	CGGTCAGCATCCTGATGGA TCAACGCTGACTTCGGAGAACT	smeDEF	(Cho et al., 2012)
*rDNA-F* *rDNA-R*	TGACACTGAGGCACGAAAGC CATCGTTTAGGGCGTGGACTA	smeDEF	(Cho et al., 2012)

### Antimicrobial Susceptibility Testing

Susceptibility of isolates to different antibiotics was evaluated according to the criteria of the Clinical and Laboratory Standard Institute (Clinical and Laboratory Standards Institute (CLSI)(2016)). Kirby-Bauer disc diffusion method was used for susceptibility testing to imipenem (10 μg), meropenem (10 μg), doripenem (10 μg), levofloxacin (5 μg), minocycline (30 μg), trimethoprim-sulfamethoxazole (1.25/23.75 μg), ceftazidime (30 μg), and tetracycline (30 μg) (Mast, Company). Minimal inhibitory concentration (MIC) was determined by MIC-Test Strip (Liofilchem; Roseto degli Abruzzi, Italy) for four selected antibiotics, including trimethoprim-sulfamethoxazole, chloramphenicol, ceftazidime, and ticarcillin-clavulanate. Quality control was performed using *E. coli* ATCC 35218 and *E. coli* ATCC 25922.

### DNA Extraction

*S. maltophilia* isolates were grown on LB for 24 h at 37°C, and genomic DNA was extracted using the high pure PCR Template Preparation Kit (Roche, Germany, and Lot.No.10362400) according to the manufacturer’s guidelines. The total DNA concentration was determined using the Nanodrop instrument (WPA Biowave II Nanospectrophotometer, USA).

### PCR-Sequencing Technique

The presence of β-lactamase genes *L1* and *L2* as well as *dfrA12*, *dfrA17*, *dfrA27*, *sul1*, *sul2*, and *Smqnr* genes were examined using the primers shown in Table 1 (Levesque et al., 1995; Hu et al., 2011, 2016; Liu et al., 2012; Kanamori et al., 2015). As described previously (Hu et al., 2011), PCR was conducted in a final volume of 25 μl containing 1 μl (20 ng) of DNA template and 12.5 μl of 2× Master Mix (SinaClon-Iran, CAT. No., PR901638), including 1× PCR buffer, 0.4 mmol/L dNTPs, 3 mmol/L MgCl_2_, and 0.08 IU *Taq* DNA polymerase, 1 μl of 10 pmol of each primer and 9.5 μl of sterile distilled water. Amplification reactions were performed on a thermal cycler (Eppendorf, Master Cycler Gradient, Germany). PCR was initiated by denaturation for 5 min 94°C, followed by 36 cycles of 45 s at 94°C, annealing at 50–59°C, according to the primers for each gene for 45 s, and extension at 72°C for 45 s. PCR products were electrophoresed by 1–1.5% agarose gel, visualized by DNA Safe staining and photographed under UV light. The PCR products were purified using a PCR purification Kit (Bioneer Co., Korea) and then, nucleotide sequencing of amplicons was performed by an ABI PRISM 3700 sequencer (Macrogen Co., Korea). The sequenced data obtained was viewed in Chromas version 1.45 software. In addition, sequence alignment was conducted using the Nucleotide BLAST program^1^.

### Phenotypic and Genotypic Detection of Biofilm Formation

Biofilm formation was examined by crystal violet staining as previously described by Stepanović et al. (2007). All experiments were performed in triplicate. An overnight culture of *S. maltophilia* was adjusted to match the turbidity of a 1.0 McFarland standard. The cultures were then diluted 1:100 in 200 ml tryptic soy broth (TSB) and were transferred into the wells of a flat-bottom polystyrene plate (SPL, Korea). After 24 h incubation at 37°C, plates were washed three times with sterile phosphate buffered saline (PBS with pH 7.3). Adherent biofilms were fixed for 60 min at 65°C, stained for 10 min at room temperature with 250 ml modified crystal violet and then, rinsed with water and allowed to dry. Biofilm samples were destained by treatment with 250 ml 33% glacial acetic acid for 20 min and the optical density (OD) was read at 492 nm (OD492). Grouping of isolates was carried out according to the following criteria: strong-biofilm producer (4 × ODc *<* OD), moderate-biofilm producer (2 × ODc *<* OD _ 4 × ODc), weak-biofilm producer (ODc *<* OD _2 × ODc), and non-biofilm producer (OD _ ODc). In addition, the presence of *rpfF*, *spgM*, and *rmlA* genes was investigated by PCR with specific primers described in Table 1 (Pompilio et al., 2011). Amplicons representing each studied gene was confirmed by sequencing analysis (Macrogen Korea). Obtained sequences were aligned in the NCBI database using BLAST program^2^.

### RNA Preparation and qRT-PCR

TMP-SMX-resistant isolates were assessed for expression of SmeDEF efflux pump. Cell suspensions were prepared and inoculated on LB broth (Cho et al., 2012). After an overnight growth, total RNA was extracted from the cell suspensions by using the RNX-Plus Kit (Cat. No., RN7713C, Sinaclon, Iran) according to the manufacturer’s instructions. The contaminating DNA was removed by RNase-free DNase I (Fermentas, USA). The total RNA concentration was determined using the Nanodrop (WPA Biowave II Nanospectrophotometer, USA). DNase-treated RNA was reverse-transcribed into cDNA using the Takara Kit (Japan). The primers used for real-time PCR are shown in Table 2. Real-time PCR assay was performed on synthesized cDNA using the Power SYBR Green PCR Master Mix (Bioneer, Korea) on a Corbett Rotor-Gene 6000 real-time rotary analyzer (Corbett Life Science, Australia). Each amplification protocol included a first denaturation step of 10 min at 94°C, followed by 40 cycles of 20 s at 94°C and 45 s at 59°C. Samples were run in triplicate. Controls run without reverse transcriptase confirmed the absence of contaminating cDNA in any of the samples. The expression level of *smeD* gene was normalized using the *rDNA* housekeeping gene, and was calculated based on 2^−ΔΔCT^ method. Results were obtained as the relative expression of the mRNA compared to that of *S. maltophilia* ATCC 13637. The parameter Ct was defined as the threshold cycle number at which the first detectable fluorescence generated by the binding of SYBR Green I dye to the minor groove of double-stranded DNA began to increase exponentially. Final results, expressed as *n*-fold differences in expression of *smeD* genes, were determined as follows:

$$\begin{array}{l} n-\mathrm{fold differences in gene expression}=\frac{Ct\;\mathrm{smeD}\;\mathrm{sample}}{Ct\;\mathrm{rDNA}\;\mathrm{sample}}/\\ \;\frac{Ct\;\mathrm{smeD}\;\mathrm{calibrator}}{Ct\;\mathrm{rDNA}\;\mathrm{calibrator}} \end{array}$$

Values of *n* < 1 were considered to indicate overexpression of the Sme efflux system.

**Table 2:** Antibiotic susceptibility of the *S. maltophilia* clinical isolates (*n* = 164).

Antimicrobial agents	MIC (μg/ml)			Disc diffusion Number (%)		
	Range	MIC_50_	MIC_90_	Susceptible	Intermediate	Resistant
Imipenem	–	–	–	–	–	164 (100%)
Meropenem	–	–	–	6 (3/65%)	–	158 (96%)
Doripenem	–	–	–	6 (3/65%)	–	158 (96%)
Ceftazidime	0.5–64	16	32	34 (20/73%)	16 (9/75%)	114 (69/51%)
Tetracycline	–	–	–	131 (79%)	–	33 (20%)
Minocycline	–	–	–	150 (91/46%)	14 (8/53%)	0 (0%)
Levofloxacin	–	–	–	163 (99/39%)	1 (0/6%)	0 (0%)
TMP/SMX	0.64– ≥ 32	0.5	≤2.38	155 (94/51%)	4 (2/43%)	5 (3/04%)
Chloramphenicol	0.5–128	16	≥32	–	–	–
Ticarcillin-clavulanate	0.5–128	16	64	–	–	–

### Molecular Typing by Multi-Locus Sequence Typing

Multi-Locus Sequence Typing (MLST) technique was performed as the same as described by Kaiser et al. (2009). Briefly, PCR for seven housekeeping genes, including *atpD*, *guaA*, *gapA*, *nuoD*, *ppsA*, *mutM*, and *recA* was carried out. Amplicons were sequenced according to the PubMLST website recommendations^3^. Unique sequence (allele) number for each gene was assigned on the basis of the information in the *S. maltophilia* MLST database^4^ to determine specific sequence type (ST). A combination of the allelic sequences of the seven genes yielded the allelic profile for each isolate.

### Molecular Typing by Repetitive Extragenic Palindromic-Pcr

Rep-PCR analyses were conducted with the single primer BoxA1R (5′-CTA CGG CAA GGC GAC GCT GAC G-3′) according to Versalovic et al. (1994). The PCR reaction mix consisted of 25 μl total volume with 12.5 μl of 2× Master Mix (Genet Bio Cat.No:G-5000) containing 1 unit of Taq polymerase in 2× reaction buffer, 10% dimethyl sulfoxide (DMSO), enzyme stabilizer, sediment, loading dye, 4 mM MgCl_2_, pH 9.0 and 0.5 mM of each dNTP, 5 μM of primer, and 1 μl of cell extract. Thermal cycling was conducted with an initial denaturation at 94°C for 10 min, followed by 25 cycles of 94°C for 45 s, 50°C for 1.5 min, 65°C for 8 min each, and concluded by a final extension of 65°C for 16 min. To determine phylogenetic relationships, rep-PCR profiles were analyzed by GelCompar II software (Applied Maths, Belgium) using the Pearson’s correlation coefficient with unweighted paired group method using arithmetic averages (UPGMA) as well as at the 80% similarity level (Adamek et al., 2011).

### Statistical Analysis

Chi-squared test was performed on the association of TMP-SMX resistance phenotype and resistance genes using SPSS software, 20.0 (SPSS Inc., Chicago, IL, USA). The Pearson’s correlation coefficient was calculated to determine the association between two variables. A significant level of *p* = 0.05 was considered statistically significant.

## Results

### Patients and Bacterial Isolates

**Figure 1:**
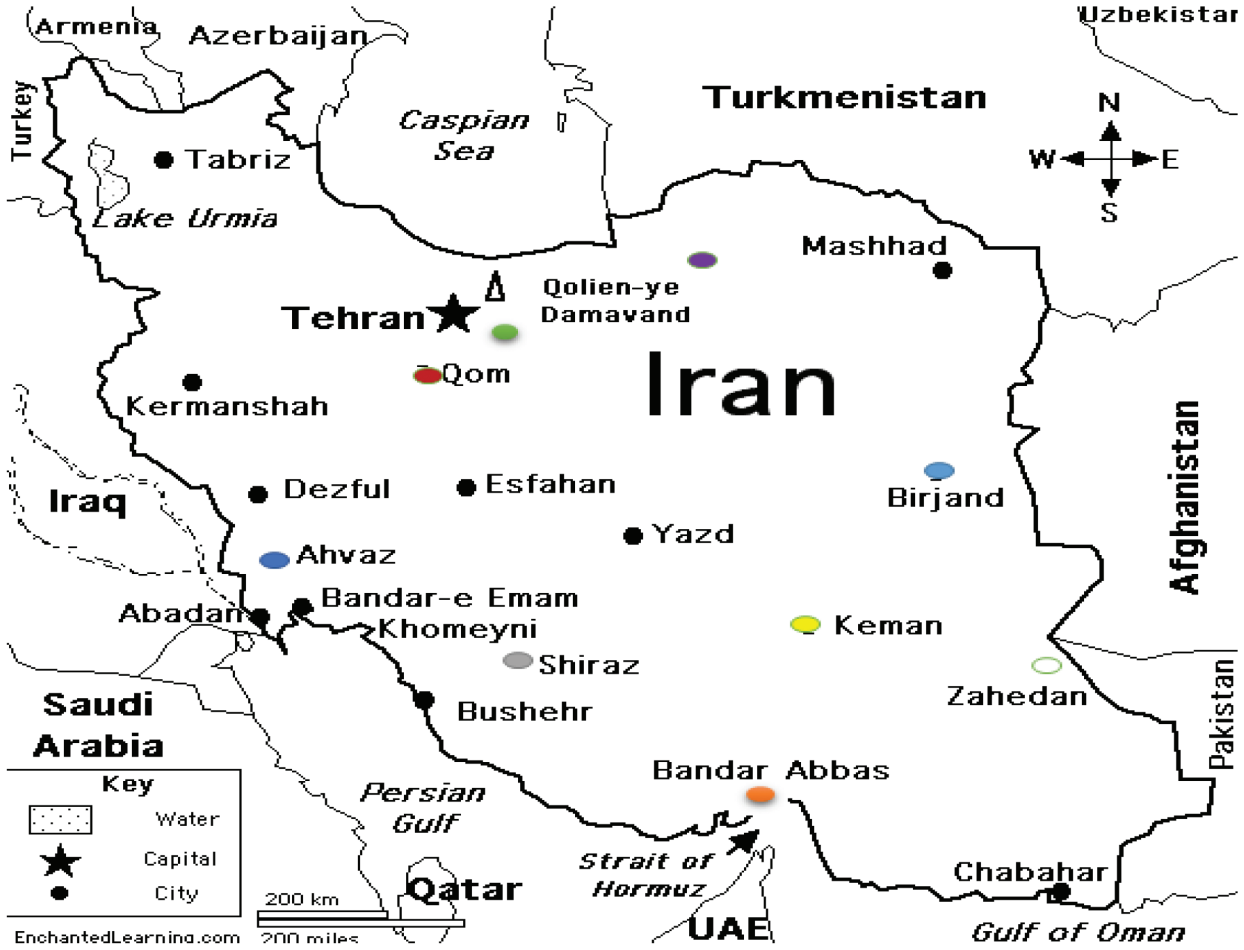
*S. maltophilia* strains isolated from Iran. 4 isolates from Birjand. 87 isolates from Tehran: Capital of Iran. 32 isolates from Ahwaz. 20 isolates from Shiraz. 14 isolates from Bandar Abbas. 4 isolates from Zahedan. 1 isolate from Kerman. 1 isolate from Gorgan. 1 isolate from Qom.

During 1-year period of study, 164 *S. maltophilia* isolates were collected from several hospitals in different regions of Iran (Figure 1).

Among the 164 isolates obtained, 88 were from males and 76 were from females (male:female ratio = 1.15). The age range of patients was from 1 month to 85 years. The majority of the isolates were originated from blood (83.53%), followed by nose/throat secretions (5.48%), cough swabs (9.75%), sputum (0.6%), and CSF (0.6%).

### Antibiotic Susceptibility Profile

Based on CLSI interpretive criteria (Clinical and Laboratory Standards Institute (CLSI) (2016)), isolates were resistant to imipenem (100%), meropenem (96%), doripenem (96%), and ceftazidime (36.58%). Interestingly, 5 (3.04%) isolates showed resistance to TMP-SMX. Minocycline and levofloxacin exhibited the highest susceptibility of 91.46 and 99.39%, respectively. The MIC ranges, MIC_50_, MIC_90_, and the percentages of isolates resistant, intermediate, or susceptible isolates to the six antimicrobial agents are shown in Table 2.

### Biofilm Phenotypes and Genotypes

**Figure 2:**
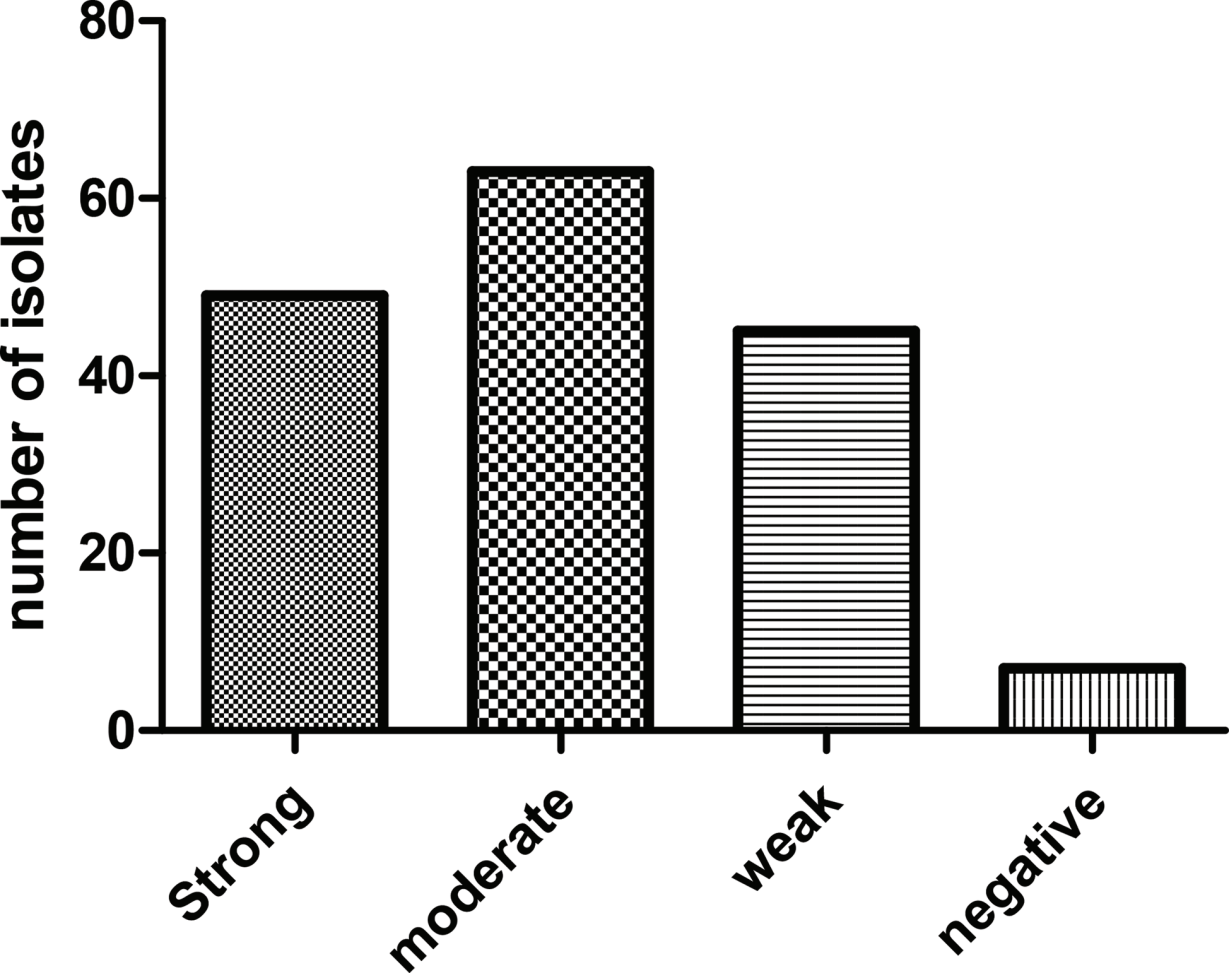
Distribution of *S. maltophilia* isolates based on the biofilm formation in crystal violet staining assay.

Biofilm phenotypes accounted for 157 out of 164 isolates (95.73%): 49 isolates (29.87%) produced strong biofilm, 63 isolates (38.41%) produced moderate biofilm, and 45 isolates (27.43%) produced weak biofilm; whereas, 7 isolates (4.26%) did not form biofilm (Figure 2). PCR-based typing of biofilm-related genes revealed an overall prevalence of 145 (88.41%), 137 (83.53%), and 164 (100%) of *rmlA*, *rpfF*, and *spgM*, respectively. In addition, the presence of *rmlA*, *rpfF*, and *spgM* had a close relationship with biofilm formation but did not significantly affect the mean amount of biofilm (*p* ≤ 0.05). Some strong- and weak biofilm-producer phenotypes had mutations within the sequence of each *rpfF*, *spgM*, and *rmlA* genes.

### Prevalence of Resistance Genes

Prevalence of resistance genes among 164 *S. maltophilia* isolates are shown in Table 3.

**Table 3:** Prevalence of resistance genes among 164 *S. maltophilia* strains isolated from Iran.

Resistance Genes, No. (%)							
*L1*	*L2*	*Smqnr*	*sul1*	*sul2*	*dfrA12*	*dfrA17*	*dfrA27*
145 (88.41%)	156 (96.12%)	103 (62.80%)	89 (54.26%)	92 (56.09%)	0 (0%)	0 (0%)	0 (0%)

Of the 145 isolates that were positive for *L1*, all 145(100%) and 139(92.3%) showed resistance to imipenem and meropenem, respectively. Amongst 156 isolates carrying the *L2* gene, all (100%) were imipenem resistant and 150 (91.1%) were meropenem-resistant (*p* ≤ 0.001). In addition, 54.19% (89/155) and 58.70% (91/155) TMP-SMX-susceptible isolates and 100% (5/5) and 20% (1/5) TMP-SMX-resistant isolates were detected to contain the *sul1*, and *sul2* genes, respectively.

### Gene Expression Analysis of *smeDEF*

Real-time PCR analysis was used to assess the expression of SmeDEF efflux system in TMP-SMX-resistant *S. maltophilia* isolates (MIC > 4/76 μg/ml). Results showed that *smeD* gene was overexpressed (5.47–7.87 fold) in two out of five isolates (40%) in comparison to the *S. maltophilia* ATCC 13637 standard strain.

### MLST Analysis

As shown in Table 4, five TMP-SMX-resistant *S. maltophilia* isolates belonged to two different STs, ST139 and ST259. This is the first report on the detection of ST139 and ST259 from Iran. In addition, ST259 (*n* = 2) with allelic profile (26, 14, 140, 103, 3, 8, 11) was not previously reported. New allele sequences were deposited at the MLST Database hosted by the Shahid Beheshti University of Medical Science, Tehran, Iran^5^.

**Table 4:** Sequence type (ST) of TMP-SMX-resistant *S. maltophilia* clinical isolates recovered in the present study.

Number of isolates	*atpD*	*gapA*	*guaA*	*mutM*	*nuoD*	*ppsA*	*recA*	ST
3	allele 3	allele 4	allele 110	allele 46	allele 6	allele 38	allele 58	139
2	allele 26	allele 14	allele 140	allele 103	allele 3	allele 8	allele 11	259

### Rep-PCR Fingerprinting

**Figure 3:**
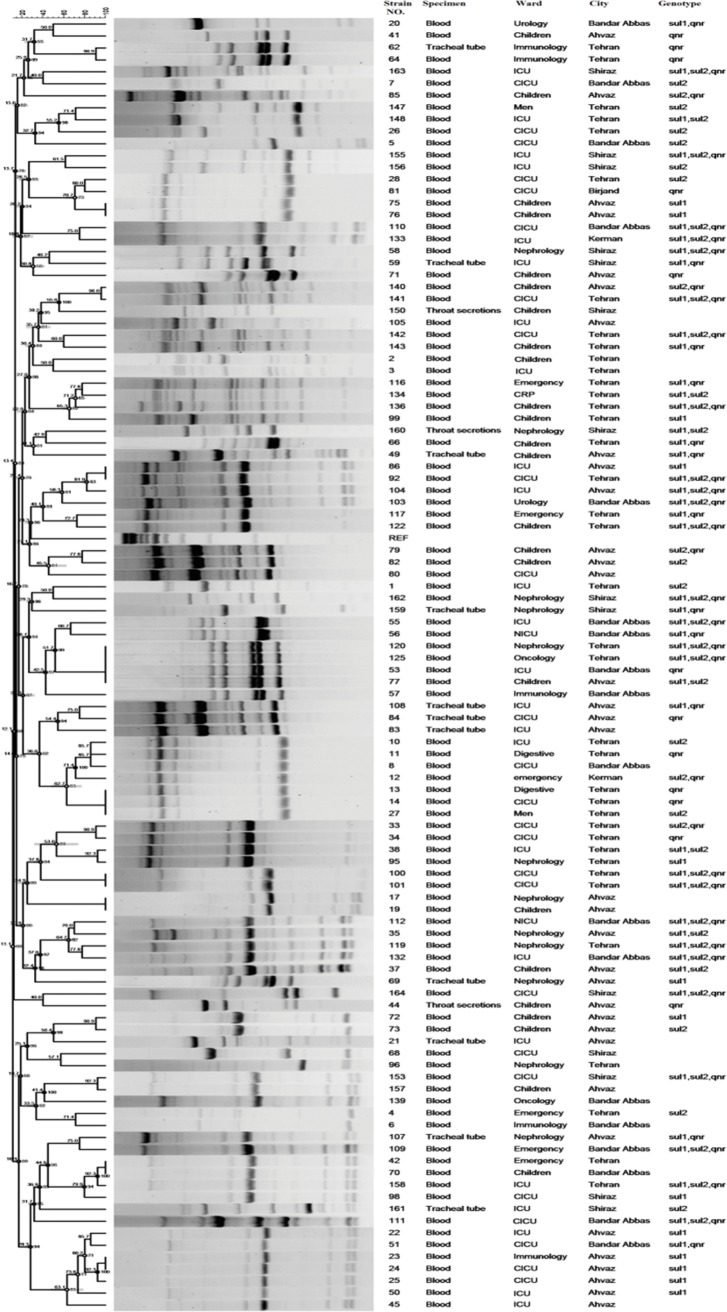
Dendrogram based on Dice’s coefficient of similarity using UPGMA method applied by the GelComparII program showing relationships between *S. maltophilia* strains according to BOX-PCR genotyping.

To evaluate the genetic diversity, all 164 *S. maltophilia* isolates were subjected to rep-PCR fingerprinting. As shown in Figure 3, isolates were divided into 16 common types (CT) containing 2–5 isolates and 114 single types (ST). Among these numerous clones, a dominant one was isolated from Ahwaz and from blood samples. The genotypic pattern of the dominant clone revealed that all isolates harbored *sul1* gene.

### Nucleotide Sequence Accession Numbers

The nucleotide sequence data reported in this study were submitted to the GenBank sequence database and assigned under the accession numbers: MF458984, MF497329, MG601517, MG640120, MG648332, MG597493, MF805867, MG640120, MG560825, MG597494, MG640119, and MG601518 for the *L1*, *L2*, *sul1*, *sul2*, *smqnR*, *atpD*, *gapA*, *guaA*, *mutM*, *nuoD*, *ppsA*, and *recA* genes, respectively.

## Discussion

The emergence of *S. maltophilia* as a nosocomial pathogen in hospitals with intrinsic resistance to multiple antibacterial agents, including carbapenems, aminoglycosides, β-lactams, and quinolones have caused great concern (Farrell et al., 2010). Additionally, some strains have acquired resistance, leading to limited antimicrobial options (Wang et al., 2013; Gholipourmalekabadi et al., 2016). In Iran, decades of misuse of antibiotics resulted in high prevalence of antibiotic resistance in bacteria (Habibzadeh, 2013; Saniee et al., 2018).

Global infectious disease surveillance stipulated that resistance rates for trimethoprim–sulfamethoxazole, ticarcillin-clavulanic acid, levofloxacin, and minocycline in *S. maltophilia* isolates are less than 4.7, 16.1, 6.5 and 5%, respectively (Sader and Jones, 2005). Among the 164 clinical isolates of *S. maltophilia* studied in the present study, a significant percentage was resistant to carbapenems (*p* ≤ 0.001). Resistance to carbapenems in *S. maltophilia* occurs through several mechanisms, including intrinsic β-lactamase expression. In this study, 145 (88.41%) and 156 (96.12%) isolates harbored L1-and L2- β-lactamase genes, respectively. Also, the results indicate that the susceptibility rate of *S. maltophilia* isolates against ceftazidime was 20.73%, with the MIC_50_ and MIC_90_ of 8 and 32 μg/ml, a figure that was in agreement with previous findings (Nicodemo and Paez, 2007). A study by Jamali et al. showed that susceptibility of *S. maltophilia* against ceftazidime was 82% with the MIC_50_ and MIC_90_ values of 2 and 32 μg/ml, respectively (Jamali et al., 2011). Shahla et al. indicated that among 11 isolate of *S. maltophilia*, 91.4% were susceptible to ceftazidime (Shahla et al., 2012). In a study by Pfaller, the susceptibility in Canada, United States, and Latin America was respectively 27, 64.7, and 93.3% and Tatmanin Turkey showed the susceptibility of 67% for this drug (Pfaller et al., 1999; Tatman-Otkun et al., 2005). A study by Farrell et al. conducted in North America, Latin America, Europe, and Asian-Pacific reported a susceptibility rate of 27.0–46.1% to ticarcillin-clavulanate among *S. maltophilia* isolates (Farrell et al., 2010). The present study showed susceptibility rate of 57.92% to ticarcillin-clavulanate. MIC_50_ and MIC_90_ for ticarcillin-clavulanate was 12 and 128 μg/ml. A study in a Brazilian hospital showed the susceptibility pattern of *S. maltophilia* against chloramphenicol differs from 11.5 to 81.4% (Nicodemo and Paez, 2007). In our study, 7.31% of isolates were found to be susceptible to this antibiotic with MIC_50_ and MIC_90_ of 24 and 64 μg/ml. This variety in results designate that the susceptibility of *S. maltophilia* is variable in different countries and even in different hospitals. Other therapeutic alternatives, such as levofloxacin and minocycline, which have been reported as effective agents for treatment of invasive *S. maltophilia* infections (Wu et al., 2012, 2013; Cho et al., 2014), showed susceptibility rates of 99.39 and 96.41% in our study. Although the prevalence of minocycline and levofloxacin-resistant *S. maltophilia* is low worldwide, continued surveillance of resistance to such antimicrobials ensures their activity.

Historically, TMP-SMX is considered the first line of defense in *S. maltophilia* infections (Chung et al., 2015; Kaur et al., 2015). Results from the SENTRY Antimicrobial Surveillance Program in 2004 indicated that 3.8% of *S. maltophilia* isolates were resistant to TMP-SMX (Fedler et al., 2006). Moreover, the resistance rate reported for Latin America, Argentina, and Malaysia were approximately less than 4.5 and 1% (Barbolla et al., 2004; Farrell et al., 2010; Neela et al., 2012). Resistance rates vary geographically but are commonly less than 10% reported in several studies (Kaur et al., 2015). However, high and different rates of resistance have been reported in patients with cancer and cystic fibrosis (Valenza et al., 2008). In different studies by Shahla et al. (2012), Hu et al. (2016), Tatman-Otkun et al. (2005), Wang et al. (2004), Nicodemo et al. (2004), and Kaur et al. (2015), the susceptibility rates were reported 47.3, 61.3, 95.8, 60, 98.6, and 22.6%, respectively. Jamali et al. showed about 60% susceptibility rate for TMP-SMX and the MIC_50_ and MIC_90_ values were 0.5 and 2 μg/ml (Jamali et al., 2011). In our study, based on the CLSI recommended dose of TMP-SMX, the resistance rate of 3.04% and the MIC_50%_ and MIC_90%_ values of 2.38 and 4.76 were found, respectively. We believe that this resistance rate for TMP-SMX, as the treatment of choice for *S. maltophilia* infection, is sustainable, making necessary the future successive reevaluation of susceptibility to this antibiotic in Iranian hospitals.

The well-known mechanism responsible for TMP-SMX resistance is harboring the *sul1*, *sul2,* and/or*dfrA* resistance genes located either on a chromosome or plasmid (Hu et al., 2011). In this study, *sul1* and *sul2* genes were detected in both TMP-SMX-resistant and TMP-SMX-susceptible *S. maltophilia* clinical isolates. Additionally, antimicrobial efflux pump mechanisms have been increasingly recognized as sources of intrinsic and acquired resistance (Song et al., 2010; Hu et al., 2011; Gholami et al., 2015). As reported in other studies, the frequency of *sul2* gene in *S. maltophilia* strains is less than that of *sul1* gene (Song et al., 2010; Hu et al., 2011). These reports are contrary to the results of our study, where a higher percentage of *sul2* and *sul1* (56.9 and 54.26%, respectively) was observed. Furthermore, both *sul1* and *sul2* genes were found in TMP-SMX -susceptible and –resistant isolates. Similar to our study, Kaur et al. indicated that the percentage of *sul1* and *sul2* were 50 and 58.3%, respectively (Kaur et al., 2015). In addition, none of the isolates tested were positive for *dfrA12*, *dfrA17*, and *dfrA27*. In contrast, a study showed that 49.1% of TMP-SMX-resistant isolates and 10.3% of TMP-SMX-susceptible isolates were positive for *dfrA* genes, among them *dfrA12* and *dfrA17* genes were more prevalent (Hu et al., 2016). Previous reports indicated that overexpression of the SmeDEF efflux system in *S. maltophilia* plays a significant role in resistance to several antibacterials, including aminoglycosides, β-lactams, and quinolones (Chang et al., 2004; Cho et al., 2012). The results showed overexpression of *smeD* in 2 (40%) of the 5 TMP-SMX-resistant clinical isolates. Sanchez et al. showed that overexpression of the SmeDEF efflux pump decreases the susceptibility to TMP-SMX (Sánchez and Martínez, 2015).

An important feature of *S. maltophilia* is its ability to form biofilms on hospital surfaces as well as on human tissues; biofilms have been related to 65% of hospital-acquired infections (Zhuo et al., 2014). In this study, the majority of isolates were biofilm-producer as well as biofilm-related gene (*rpfF*, *rmlA* and *spgM*) carrier. In a study by Flores-Trevino et al., they showed that all *S. maltophilia* isolates were able to form biofilm and 47.9, 38.7, and 13.4% of the isolates were weak-, moderate-, and strong-biofilm producers, respectively (Flores-Trevino et al., 2014). Zhou et al. showed that the results of a biofilm formation assay on polystyrene was strong in 49 (29.87%) strains, moderate in 63 strains (38.41%), and weak in 45 (27.43%) strains, while nine strains (4.26%) were non-biofilm former. Furthermore, the presence of *rpfF* and *spgM* was significantly correlated to biofilm formation. Pompilio et al. reported that *spgM* gene played a significant role in formation of strong biofilm among *S. maltophilia isolates* (Zhuo et al., 2014). Similarly, the presence of *rmlA*, *rpfF*, and *spgM* genes in the present study improved significantly biofilm formation by *S. maltophilia* isolates tested (*p* ≤ 0.05). Indeed, the isolates with *rpfF*^+^/*spgM*^+^/*rmlA*^+^ genotype were associated with production of moderate or strong biofilm. In addition, amino acid substitution in genes encoding SpgM, RpfF and RmlA were found among some strains (Corlouer et al., 2017). However, it is still unclear which gene mutation results to change in biofilm formation.

High genetic diversity among *S. maltophilia* isolates has been described worldwide. Although occurrences of outbreaks within hospital settings have also been reported (Flores-Trevino et al., 2014). Recently, molecular epidemiologic studies, like MLST is developed for *S. maltophilia* strain-typing that focuses on conserved housekeeping genes (Corlouer et al., 2017). In the present study, MLST analysis was performed for determining genetic diversity of five TMP-SMX-resistant isolates. The results revealed two STs (ST139 and ST259), of which ST259 was identified for the first time in this study. Similarly, studies in Spain in 2004, and Korea in 2010, a high rate of genetic diversity among *S. maltophilia* isolates despite their source in a single hospital (Valdezate et al., 2004; Cho et al., 2012; Corlouer et al., 2017). These findings indicate that *S. maltophilia* has a high potential for environmental distribution, although database analysis shows that there are noticeably fewer STs for *S. maltophilia* isolates than other bacterial isolates. Rep-PCR fingerprinting is a method with lower cost and the best time efficiency. According to the cluster analysis of *S. maltophilia* strains, this study detected high clonal diversity among the isolates. The only exception is the dominant common type including strains isolated from blood culture of patients hospitalized in Ahwaz. In addition, all these isolates harbored *sul1* gene. As a result, the presence and spread of these strains with resistance gene could be a significant threat.

## Conclusions

This multi-institutional study revealed that *S. maltophilia* is an emerging MDR opportunistic pathogen in hospital settings, especially among immunocompromised patients. TMP-SMX remains the most effective antibacterial agent against *S. maltophilia*. So, a significant effort is required to maintain antibacterial properties of this antibiotic. Due to the low prevalence of resistance to two antibiotics levofloxacin and minocycline, clinical usage of these agents can be continued. The findings of this study showed an increasing presence of antibacterial resistance-and biofilm genes among the clinical isolates of *S. maltophilia* strains in Iran. Clinicians must consider that *S. maltophilia* as a co-pathogen or co-colonizer in polymicrobial infections can have negative effect on the success rate of antibacterial treatment and clinical outcome. In our opinion, this is significant medical problem, which should be of great concern.

## Author Contributions

NB, ZG, FF, and AH conceived and designed the experiments. NB, ZG, AH, AY, JM, AA, SS, ST, AA, FF, and AP performed the experiments and analyzed the data. NB, AA, and AH wrote the paper.

### Conflict of Interest Statement

The authors declare that the research was conducted in the absence of any commercial or financial relationships that could be construed as a potential conflict of interest.

## Footnotes

^1^http://www.ncbi.nlm.nih.gov/nucleotide/

^2^http://www.ncbi.nlm.nih.gov/nucleotide/

^3^http://pubmlst.org/smaltophilia/

^4^http://pubmlst.org/smaltophilia/

^5^http://pubmlst.org/perl/bigsdb/bigsdb.pl?db=pubmlst_smaltophilia_isolates&page=query
